# Self-other distinction modulates the social softness illusion

**DOI:** 10.1007/s00426-021-01549-8

**Published:** 2021-06-25

**Authors:** Maria Pyasik, Elisabetta Fortunato, Olga Dal Monte, Selene Schintu, Francesca Garbarini, Tommaso Ciorli, Lorenzo Pia

**Affiliations:** 1 SAMBA (SpAtial, Motor and Bodily Awareness) Research Group, Department of Psychology, University of Turin, Turin, Italy; 2 NPSY-Lab.VR, Department of Human Sciences, University of Verona, Verona, Italy; 3 Department of Psychology, University of Turin, Turin, Italy; 4 Department of Psychology, Yale University, New Haven, CT USA; 5 Department of Psychology, George Washington University, Washington, DC USA; 6 MANIBUS - Movement ANd Body In Behavioral and Physiological neUroScience Research Group, Department of Psychology, University of Turin, Turin, Italy; 7 NIT (Neuroscience Institute of Turin), Turin, Italy

## Abstract

The social softness illusion (i.e., the tendency to perceive another person's skin as softer than our own) is thought to promote the sharing of social-emotional experiences because of the rewarding properties of receiving and giving social affective touch. Here we investigated whether the ability to distinguish someone else’s body from our own modulates the social softness illusion. In particular, we tested whether the spatial perspective taken by the participants and seeing or not the touched arms could alter this illusion. Pairs of female participants were assigned the roles of either the giver (i.e., delivering the touches) or the receiver (i.e., being touched). We manipulated the location of the touch (palm or forearm), the spatial perspective of the receiver’s body with respect to the giver’s body (egocentric or allocentric perspective), and the vision of the touched body part (the giver could either see both her own and the receiver’s body part, or she was blindfolded). Consistently with previous findings, the skin of another person was perceived as softer than the own one. Additionally, the illusion was present for both the forearm and the palm, and it was stronger in allocentric compared to the egocentric perspective (i.e., when the self-other distinction was clearer). These findings show that the mechanisms underpinning the ability to represent another person’s body as distinct from our own modulates the social softness illusion, and thus support the role of the social softness illusion in fostering social relationships.

**Supplementary Information:**

The online version contains supplementary material available at 10.1007/s00426-021-01549-8.

## Introduction

Touch is a cornerstone of human nature, being fundamental in cognition, emotions and social interactions (Field, 2003; Gallace & Spence, 2010). Beyond the classical discriminative system (Penfield & Boldrey, 1937), recent evidence pinpointed the existence of a hyper-specialized neurophysiological somatosensory system, called CT-afferent system (Loken et al., 2009). This system processes social affective, rather than discriminative, properties of touch and is specifically activated by touches that fully resemble the human-to-human caressing, which is typical of intimate relationships, and characterized by slowness and lightness (McGlone et al., 2014; Olausson et al., 2010).

The strong hedonic nature of the affective touch brought to the forefront of psychological sciences a fascinating interpretation of this phenomenon. The affective touch would have the specific and distinctive evolutionary meaning of constructing and sustaining interpersonal relationship (Morrison, 2016). One of the most compelling pieces of evidence supporting this claim is the social softness illusion, recently discovered by Gentsch and colleagues (Gentsch et al., 2015). With a series of experiments, the authors demonstrated that the skin of another person is perceived as being softer than our own skin, regardless of its actual softness. Interestingly, the social softness illusion was successfully induced for the forearm (i.e., the hairy skin) but not for the palm (i.e., the glabrous skin). The illusion has been interpreted as a useful mechanism to create social bonds by enhancing the motivation to touch others and engage in intimate relations. In other words, humans would structure and maintain their social ties also because of the rewarding properties of giving and receiving affective touch.

Social interactions require a solid self-other distinction and any successful interpersonal exchange through touch necessarily entails the representation of another person’s body as distinguished from one’s own (i.e., sense of body ownership). Such ability is strongly grounded in spatial reference frames, since others’ bodies are framed in an allocentric perspective, whereas the own one is represented in an egocentric perspective (Cleret de Langavant et al., 2011; Degos et al., 1997). The ability to adopt an allocentric perspective can be considered an embodied cognitive process that enables the understanding of others’ mental state and facilitates social interactions (Kaiser et al., 2008; Kessler & Thomson, 2010). Moreover, representing another person’s body as distinguished from the own body relies on processing both motor and sensory signals concerning the physical body [e.g., (Burin et al., 2017a, 2017b; Kalckert & Ehrsson, 2017; Pyasik et al., 2018; Rognini et al., 2013; Romano et al., 2013; Tsakiris et al., 2006)]. With respect to sensory modalities (i.e., touch, proprioception, vision and audition) an optimal integration among those different sources allows to build a clear representation of the own body and, thus, promotes the self-other distinction (Tsakiris, 2017). Among the human senses, vision seems to have a dominant role, supporting the idea of a stronger contribution to this process (Tsakiris, 2017). Summarizing, these considerations suggest that spatial perspective and vision are useful mechanisms to maintain the representation of others’ bodies as physically distinct from our own body, a critical element in social relationships. Hence, here we tested whether these two variables could modulate the social softness illusion, interpreted as a useful mechanism to create social bonds, and whether this could be related to the type of skin stimulated (hairy or glabrous skin). We hypothesized that the social softness illusion would occur more strongly when the self-other distinction was the most vivid, namely, when the other person’s body part was touched in an allocentric versus egocentric perspective and when the other’s hand was visible during touching. In line with Gentsch and colleagues’ study (Gentsch et al., 2015), we expected the social softness illusion to occur for the hairy skin (the forearm) but not for the glabrous skin (the palm).

## Methods

### Participants

Based on a priori power analysis conducted in G*Power (Faul et al., 2007) for 2 × 2 × 2 repeated measures ANOVA with a medium effect size (f = 0.25) and alpha level = 0.05, the minimal required sample size for reaching the power of 0.80 was determined to be sixteen.

Twenty-four right-handed (Oldfield, 1971) participants (age range – 20–28 years) were recruited in our study to account for possible dropouts. All participants, without any history of neurological or psychiatric disorders, gave written informed consent to participate in the study. As in Gentsch and colleagues’ study (Gentsch et al., 2015), we included only female participants to avoid significant variations in skin appearance (e.g., higher density of hair) and possible gender differences in the perception of social touch. Furthermore, none of the included participants had any skin abnormalities, such as scars or tattoos, on the palm and forearm area and all of them were naïve to the purpose of the study.

All experimental procedures were approved by the Bioethical Committee of the University of Turin and conducted in accordance with the ethical standards of the 2013 Declaration of Helsinki.

### Experimental design

A modified version of the ‘touch protocol’ adopted from Gentsch and colleagues’ study (Gentsch et al., 2015) was used. Pairs of female participants were randomly assigned to be either the giver (i.e., touching the other participant) or the receiver (i.e., being touched by the other participant). The roles were reversed after the first session, thus providing a within-subject design. We manipulated three variables: the location of the touch (the palm, i.e., glabrous skin, or the forearm, i.e., hairy skin), the vision of the touched body part (the giver could either see both her own and that receiver’s body part, or she was blindfolded), and the position with respect to the giver’s body (egocentric or allocentric).

**Fig. 1:**
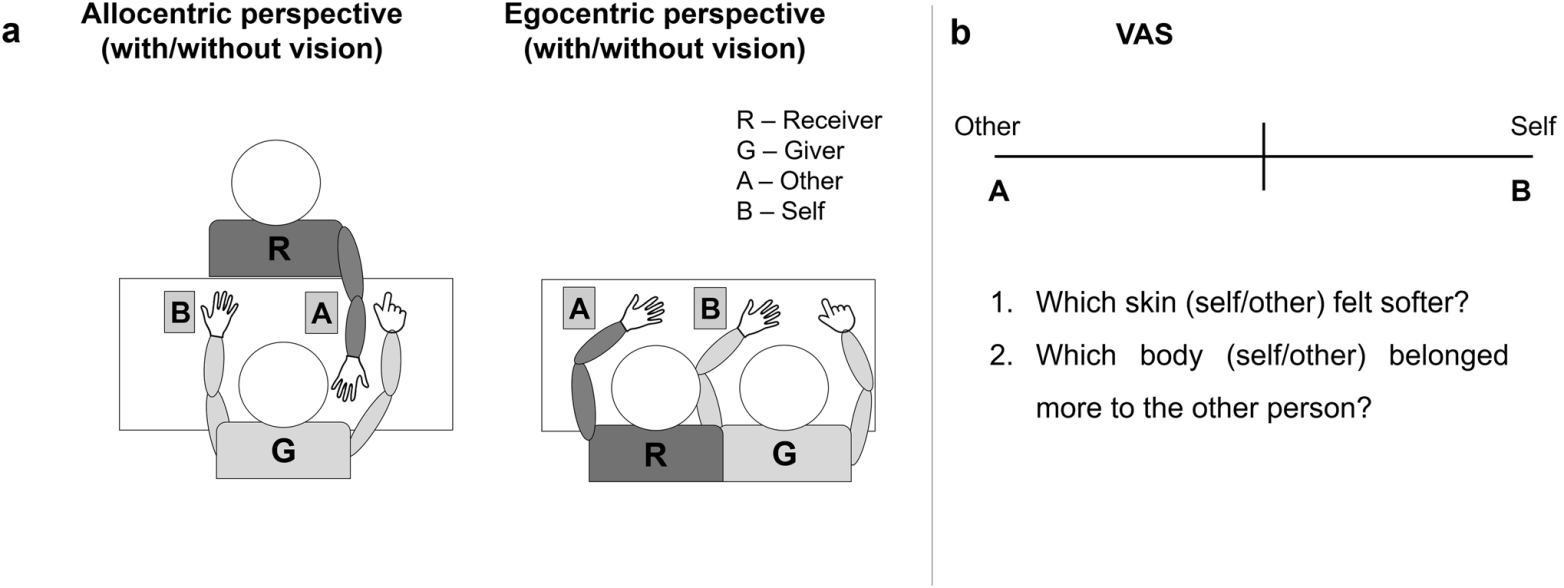
Experimental setup and procedure: **a** Experimental setup depicting the two different perspectives: allocentric on the left and egocentric on the right. The letter G indicates the giver participant, the letter R indicates the receiver participant. The giver-participant was instructed that the letter A represented the other participant’s body and B represented her own body. These letters were placed near to the receiver and to the giver, respectively, and used during the VAS ratings; **b** Visual Analogue Scale (VAS) and questionnaire statements. Negative values indicated attribution to the other participant and positive values indicated attribution to self

In the egocentric condition (Fig. 1a) the two participants sat next to each other at the same side of the table and placed their left arms and hands on the table parallel to each other approximately 30 cm apart and with a 45 ° angle with respect to the giver participant’s body. The giver participant was instructed to keep her right hand on the table, while the receiver participant was asked to keep it out of the view (under the table, placed on her lap).

In the allocentric condition (Fig. 1a), the two participants sat in front of each other at the opposite sides of the table and both placed their left arms and hands on the table in front of them approximately 30 cm apart. As in the egocentric condition, the giver participant kept her right hand on the table and the receiver participant kept it out of the view. In both conditions, the giver participant was facing a computer screen, located approximately 80 cm away, displaying the visual analogue scale (VAS), which was used for measuring both softness of the skin and sense of body ownership throughout the task (see Procedure section for details).

### Procedure

Before the beginning of the experimental task, the areas of touch (approximately 9 × 4 cm) were marked on both participants’ forearms and palms. The giver participant was trained to lightly touch her palm and forearm (self-touch) or the receiver participant’s palm and forearm (other-touch). The touch was performed with the right hand (with index, middle and ring fingers) and executed in form of stroking movements with a speed of approximately 3 cm/s, i.e., the speed that is known to promote the affective touch (Löken et al., 2009). To maintain a constant stroking speed, the giver participant followed a digital metronome. Participants were then trained to automatize the procedure.

The experimental task consisted of eight types of trials that were combined according to three factors: (1) location of the touches (forearm/palm); (2) position of receiver participant’s hand (egocentric/allocentric with respect to giver participant’s body); (3) presence/absence of a vision of the touched body parts. In addition, in each trial type, the order of the touches (self-touch and other-touch) was alternated (see Table 1 for the summary of the trials). In each trial, the giver participant was instructed to perform the touches for 12–6 s (i.e., two successive strokes) for self-touch and 6 s (i.e., two successive strokes) for other-touch, or vice versa. The sound of the metronome indicated the beginning of each trial. The experimenter ensured that participants, as instructed, were avoiding any communication or eye contact during the experiment.

**Table 1:** Summary of trial types used in this study. Each trial was composed of one Self-touch and one Other-touch

Self-touch (palm, egocentric, vision) → Other-touch (palm, egocentric, vision)
Self-touch (palm, allocentric, vision) → Other-touch (palm, allocentric, vision)
Self-touch (palm, egocentric, no-vision) → Other-touch (palm, egocentric, no-vision)
Self-touch (palm, allocentric, no-vision) → Other touch (palm, allocentric, no-vision)
Other-touch (palm, egocentric, vision) → Self-touch (palm, egocentric, vision)
Other-touch (palm, allocentric, vision) → Self-touch (palm, allocentric, vision)
Other-touch (palm, egocentric, no-vision) → Self-touch (palm, egocentric, no-vision)
Other-touch (palm, allocentric, no-vision) → Self-touch (palm, allocentric, no-vision)
Self-touch (forearm, egocentric, vision) → Other-touch (forearm, egocentric, vision)
Self-touch (forearm, allocentric, vision) → Other-touch (forearm, allocentric, vision)
Self-touch (forearm, egocentric, no-vision) → Other-touch (forearm, egocentric, no-vision)
Self-touch (forearm, allocentric, no-vision) → Other-touch (forearm, allocentric, no-vision)
Other-touch (forearm, egocentric, vision) → Self-touch (forearm, egocentric, vision)
Other-touch (forearm, allocentric, vision) → Self-touch (forearm, allocentric, vision)
Other-touch (forearm, egocentric, no-vision) → Self-touch (forearm, egocentric, no-vision)
Other-touch (forearm, allocentric, no-vision) → Self-touch (forearm, allocentric, no-vision)

We varied 3 variables: Location (Palm and Forearm), Perspective (Egocentric and Allocentric) and Vision (Vision and No-vision). We had 16 different trials and each trial was repeated twice, for a total of 32 trials for each subject

Following each trial, the giver participant was asked to make comparative judgments between self-touch and other-touch using a VAS scale to quantify whose skin (self or other) felt softer [“Which skin (self/other) felt softer?”]. Given that distinguishing the softness of one’s own skin from the one belonging to another person requires a strong sense of body ownership, that is the feeling that your palm or forearm unambiguously belongs to yourself (Gallagher, 2000), we additionally quantified participants’ body ownership with another VAS scale [“Which body (self/other) belonged more to the other person?”]. For both VAS scales (softness and sense of body ownership), the giver participant was asked to respond by moving a cursor (controlled by a computer mouse) to the position on the VAS that corresponded the most with her response. The VAS (Fig. 1b) was a 20 cm long horizontal line having the left and right poles marked as “A” and “B”, respectively. As shown in Fig. 1a, two pieces of paper were placed near the participants’ left hands; the one marked with an “A” was placed near the receiver participant’s hand and the one marked with a “B” ‒ near the giver participant’s hand; no other words or numbers were present on the VAS. The giver participant was instructed that A represented the other participant’s palm/forearm and B represented her own palm/forearm (hence, negative values indicated attribution to the other participant and positive values indicated attribution to oneself). The order of the VAS questions (softness and sense of body ownership) were randomized in each trial, to control for any potential rating bias due to the order presentation.

Each trial was repeated twice for each of the four conditions, which resulted in eight trials per condition and thirty-two trials (i.e., data points) in total per participant. The order of experimental conditions and trials was defined according to the Latin square. After the completion of the experimental task, the participants’ roles were reversed, and the task was repeated after a short break. The between-subject design allowed us to control for physical skin differences between participants.

### Statistical analysis

In each condition, the VAS scores for each of the two questions were averaged between trials. All variables were tested for normality of distribution using Shapiro–Wilk test and residual errors of the ANOVAs were evaluated with QQ plots, and since none of the variables violated the criteria of normality, parametric tests were used.

First, to test whether our sample experienced a significant attribution of softness/ownership towards their own or other’s palm/forearm, we performed two one-sample t tests on the VAS ratings against zero (i.e., no difference between own and other’s body part) for the softness and ownership scores averaged across all conditions. Then, we compared the VAS ratings for softness and ownership between conditions. The ratings (separately for softness and ownership) were analyzed with a repeated-measures ANOVA having Location (forearm, palm), Perspective (egocentric, allocentric) and Vision (no-vision, vision) as within-subject factors. In the case of multiple post hoc comparisons, the p values were corrected with Newman-Keuls test. Effect sizes were reported as Cohen’s *d* or η_p_^2^.

## Results

**Fig. 2:**
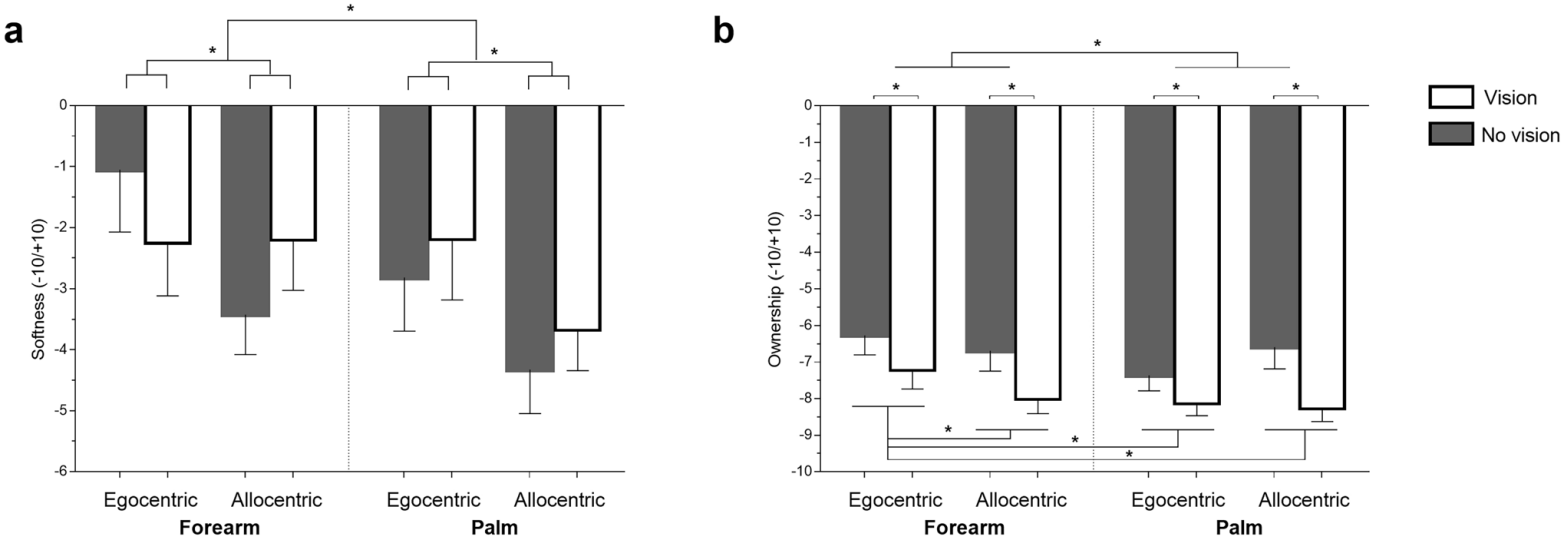
The social softness illusion: **a** Softness ratings (−10/ + 10); **b** Ownership ratings (−10/ + 10). Negative values indicated attribution to the other participant (ratings towards the letter “A” on the VAS that represented the receiver participant) and positive values indicated attribution to oneself (ratings towards the letter “B” on the VAS that represented the giver participant herself). Error bars represent standard error of means; ^*^significant differences

As regards the softness ratings, the one-sample t test against zero [t(23) = −4.38, *p* < 0.001, d_z_ = 0.89] showed the presence of the social softness illusion, meaning that the other’s skin was rated as significantly softer than one’s own across conditions. The repeated measures ANOVA having Perspective (egocentric, allocentric), Location (forearm, palm), and Vision (no-vision, vision) as between-subjects variables revealed a significant main effect of Perspective [F(1,23) = 7.72, *p* = 0.01, η_p_^2^ = 0.25], with stronger attribution of softness to other in allocentric (mean ± SEM = −3.41 ± 0.58) perspective compared to egocentric (mean ± SEM = −2.08 ± 0.75), and a significant main effect of Location [F(1,23) = 4.45, *p* = 0.05, η_p_^2^ = 0.16], with stronger softness illusion for the palm (mean ± SEM = −3.26 ± 0.64) compared to the forearm (mean ± SEM = −2.24 ± 0.70; see Fig. 2a). There was no significant main effect of Vision, or any other second and third-order interactions (all F ≤ 3.19, *p* ≥ 0.09). Thus, we found that overall participants experienced the social softness illusion, but that it was stronger in the allocentric compared to the egocentric perspective, and the other’s palm was rated softer than the forearm.

With respect to the ownership ratings, the one-sample t test against zero [t(23) =  −21.57, *p* < 0.001, d_z_ = 4.40] revealed a significant attribution of ownership to the receiver participant, meaning that the participants always identified other’s body parts as belonging to the other person. The repeated measures ANOVA having Location (forearm, palm), Perspective (egocentric, allocentric) and Vision (no-vision, vision) as between-subjects variables revealed a significant main effect of Location [F(1,23) = 5.50, *p* = 0.03, η_p_^2^ = 0.19], with stronger other-attribution (i.e., more negative ratings) for the palm (mean ± SEM = −7.59 ± 0.32) than for the forearm (mean ± SEM = −7.05 ± 0.40), and a significant main effect of Vision [F(1,23) = 13.92, *p* = 0.001, η_p_^2^ = 0.38] where the other-attribution was stronger with vision (mean ± SEM = −7.92 ± 0.32) conditions compared to no-vision (mean ± SEM = −6.73 ± 0.43). Moreover, we found a significant Location x Perspective interaction [F(1,23) = 6.13, *p* = 0.02, η_p_^2^ = 0.21]. Post hoc comparisons revealed that despite all the givers discriminated well their body from the receiver’s one, when the giver was in egocentric perspective she attributed the receiver’s forearm less to the other person (mean ± SEM = −6.75 ± 0.45) compared to other conditions [forearm allocentric (p_corr_ = 0.03; mean ± SEM = −7.36 ± 0.39), palm egocentric (p_corr_ = 0.005; mean ± SEM = −7.75 ± 0.33), palm allocentric (p_corr_ = 0.04; mean ± SEM = −7.44 ± 0.37); Fig. 2b). All the other comparisons were not significantly different from each other (0.24 ≤ p_corr_ ≤ 0.76), and there was no evidence of a higher-order interaction involving any of these factors (all F ≤ 1.51, *p* ≥ 0.23). Summarizing, the attribution of the body parts to another person was stronger for the palm than for the forearm, and for the latter body part, it was weaker in the egocentric perspective compared to the allocentric one (i.e., when the distinction between self and other is less clear). Moreover, the other attribution was stronger with the presence of vision independently of body parts and perspective.

## Discussion

In the present study, we examined whether the perspective taken by the participants, and the vision of the touched body parts modulated the social softness illusion. Participants lightly touched their own and the other person’s forearm and palm, located either in an egocentric or an allocentric perspective, while they were able to see or not both body parts. At the end of each trial, the touch giver was then asked to rate which skin (self or other) felt softer and which body part (self or other) belonged more to the other person.

Overall, we found that the skin of the other participant’s, on both forearm and palm locations, was rated as softer than the own one (i.e., the social softness illusion), and the other’s participant body was attributed more to the other person than to the self (i.e., sense of ownership). These findings not only confirmed the existence of the social softness illusion as reported by Gentsch and colleagues (Gentsch et al., 2015) but also showed that the other person’s body was clearly perceived as distinguished from the own body (i.e., the sense of body ownership). We found, however, that the illusory effect was present not only for the forearm (Gentsch et al., 2015) but also for the palm, with the latter being also the location where the distinction between one’s own and other’s body was greater. It is known that unmyelinated, small-diameter, lower-threshold mechanoreceptive afferents (i.e., CT afferents) involved in the transmission of affective aspects of touch are present only in hairy skin, which would explain the presence of the softness illusion for the forearm. Nonetheless, several studies have reported that people rate touch to the palm as pleasant as the one to the arm (Klocker et al., 2014; Loken et al., 2009; McGlone et al., 2012, 2014; Pawling et al., 2017). Affective touch on the glabrous skin touch, in which CT afferents are absent, activates some of the brain structures overlapping with those related to the touch of the hairy skin, as well as some unique ones (Gordon et al., 2013). McGlone and colleagues (McGlone et al., 2012) reported that applying a gentle and affective touch to the forearm (the hairy skin) activated the posterior insula and orbito-frontal cortex to a greater extent than the touch to the palm, whereas a touch to the palm increased the activity of the somatosensory cortex. This differential activation reinforces the idea that CT fibers support the innate rewarding value of touch, whereas Aβ fibers subserve a learned reinforcement mechanism based on previous and remembered pleasant tactile experience (McGlone et al., 2012, 2014). Consistently, it has been reported that touch on hairy skin is assigned more emotional/affective verbal descriptors, whereas the one on the palm more sensory descriptors (Ackerley et al., 2014; McGlone et al., 2012). It is also worth noticing that most of the literature about affective touch has investigated the effects and mechanisms from the perspective of the receiver, and little is known about the psychophysiology and neural mechanisms from the perspective of the giver. To sum up, our findings showing the presence of the softness illusion also for the palm are compatible with the fact that, so far, the differential role of hairy and glabrous skin in the emergence of the social softness illusion is not fully understood.

The main aim of our study was to manipulate both the participant’s spatial perspective and the vision of the two body parts during the social softness illusion. We found that the spatial perspective affected both softness and ownership ratings. The allocentric, as compared to the egocentric, perspective increased both the perceived softness of the other participant’s skin and attribution of that body part to the other person (in particular, when the palm was touched). In other words, the illusion increased when the other person’s body was framed in the perspective typical of perceiving other bodies, as compared to the perspective in which the own body is experienced. It is therefore possible that the self-other distinction for both the forearm and the palm was modulated by the position of the other’s arm: being enhanced in the allocentric perspective and thus increasing the perceived softness of the other person’s skin. This is in line with the existing accounts for body ownership: as mentioned above, one of the principal top-down constraints of body ownership (e.g., in the case of the rubber hand illusion) is the anatomical congruence of the embodied limb/body (Tsakiris, 2010). Indeed, it has been shown that, if the fake hand is placed incongruently with the participant’s body, i.e., in the allocentric perspective, the illusory ownership is clearly absent (Burin et al., 2017b; Costantini & Haggard, 2007; Pyasik et al., 2021). Furthermore, this constraint might be even more important than the synchrony of multisensory information in the egocentric perspective, as some studies have shown a certain degree of embodiment even with asynchronous stimulation of the fake hand (Costantini et al., 2016; Pyasik et al., 2020). What is the possible contribution of perspective on the social softness illusion? As already mentioned, the CT-afferent system encodes the pleasure of touch and, consequently, it deeply affects social relationships from the receiver point of view (Hertenstein et al., 2006). Nonetheless, social affective touch is almost always reciprocal and then, it must necessarily also influence the giver. Since softness and smoothness are highly rewarding tactile attribute (Kida & Shinohara, 2013), the social softness illusion might represent an automatic mechanism that reinforce the giver’s behavior by which 'giving pleasure is receiving pleasure’. Such interpretation seems to be consistent with the abovementioned higher softness rating for the palm than for the forearm that, indeed, was entirely triggered by the allocentric perspective (i.e., the difference disappeared if the analysis was limited to the egocentric perspective, the only one employed by Gentsch and colleagues (Gentsch et al., 2015)). It is known that pleasant touch can be felt where the CT fibers are not present (i.e., on the palm), meaning that also the Aβ fibers (denser in glabrous than hairy skin (Boada et al., 2010)) process the affective components of touch. However, the role of these fibers is more a learned association between the stimulus and the pleasant context in which it has been experienced (McGlone et al., 2014). Hence, shifting the position of the giver towards a perspective in which interpersonal interactions through touch have been previously experienced (i.e., allocentric) might increase the role of the Aβ fibers in perceiving pleasant touch (and enhance the illusory effect for the palm). All in all, the social softness illusion could be conceived as an unconscious representation in the givers’ brain that includes the features that most likely elicit pleasure in another person according to previous experiences. Some of these critical features might be the touch stroking speed and the perspective in which the other person’s body is typically framed.

As regards the variable vision, it did not affect the softness ratings but affected the ownership ones. In other words, being able to see the touched body part did not increase the perceived softness of the other participant’s skin but increased the attribution of that body part to the other person. Body ownership is known to rely on the optimal spatiotemporal integration among sensorimotor signals (Tsakiris, 2017). Evidence is gathered from the rubber hand illusion paradigm in which perceiving tactile stimuli delivered on the own (hidden) hand, but concurrently seen on the rubber hand located in an anatomically congruent posture, induces the illusory ownership of the artificial hand (Burin et al., , 2017b, 2018; Costantini et al., 2016; Kalckert & Ehrsson, 2014; Kammers et al., 2011; Longo et al., 2008; Pyasik et al., 2020; Tsakiris & Haggard, 2005). Interestingly, however, it has been demonstrated that body ownership illusion can emerge through vision alone, without any concurrent tactile stimulation (Ferri et al., 2013; Rohde et al., 2011). Similarly, it has been recently discovered that a category of neurological patients can embody somebody else’s arm by simply seeing that arm (Pia et al., , 2016, 2020). Taken together, these data indicate that vision is perhaps the most relevant sense for body ownership and, thus, contributing to a clear self-other distinction. Capitalizing on findings showing that the view of caressing triggers similar brain activities as directly felt caressing touch (Morrison et al., 2011), we predicted that seeing the two body parts might have enhanced the social softness illusion. However, exactly as Gentsch and colleagues (Gentsch et al., 2015) we did not find such effect. It rather appears that the affective value of touch is what drives the social softness illusion regardless of whether visual information is concurrently available.

One might argue that a possible limitation of the study is the fact that even though the distance between the touched arms (giver and receiver) was the same across perspectives, in the allocentric one the receiver’s arm was closer to the giver’s hand that performed the touches (while in the egocentric perspective it was the opposite). This might have influenced the illusion to some extent. However, the possible effect of perspective was controlled by the fact that each trial was repeated twice with alternating order of touches, i.e., within each condition the giver participant performed the self-touch first, followed by the touch of the other’s arm, and in the second trial, the other-touch was performed first followed by the touch to own arm. Importantly, Gentsch and colleagues (Gentsch et al., 2015) directly tested the possible influence of the proximity of the touched body part in the social softness illusion and found no significant effects.

In summary, our results suggest that the processes underpinning self-other distinction modulate the social softness illusion. This, in turn, supports the idea that the mechanisms subserving the softness illusion could be useful to create social bonds and that affective touch supports the human ability to socialize. Moreover, these findings support the evidence that pleasantness of touch not only relies on bottom-up incoming sensory signals but also on contextual factors (Ellingsen et al., 2014; Gazzola et al., 2012). However, it is important to note that very little is known about the contribution, similarities, and differences of the Aβ and CT fibers to touch and emotional processing in general. Future studies should further investigate whether the skin that is perceived softer by the giver is also the skin with higher CT fibers. Along the same line, it seems critical to better understand whether affective touch is perceived as pleasant for the giver as it is for the receiver, measuring the sense of pleasantness simultaneously for both receiver and giver. Future work might also further investigate the contribution that the sense of ownership (distinction between self and other) might have on the affective touch and, more broadly, on the construction of interpersonal relationships.

## Supplementary Information

Below is the link to the electronic supplementary material.

Supplementary file1 (XLSX 12 kb)

## Data Availability

All data generated and analyzed in this study are available in the manuscript and the Supplementary Materials.

## Code availability

The code is available on request to the corresponding author.
